# MRI for Assessing Response to Neoadjuvant Therapy in Locally Advanced Rectal Cancer Using DCE-MR and DW-MR Data Sets: A Preliminary Report

**DOI:** 10.1155/2015/514740

**Published:** 2015-08-27

**Authors:** Mario Petrillo, Roberta Fusco, Orlando Catalano, Mario Sansone, Antonio Avallone, Paolo Delrio, Biagio Pecori, Fabiana Tatangelo, Antonella Petrillo

**Affiliations:** ^1^Division of Radiology, Department of Diagnostic Imaging, Radiant and Metabolic Therapy, Istituto Nazionale Tumori-IRCCS “Fondazione Giovanni Pascale”, Via Mariano Semmola, 80131 Naples, Italy; ^2^Department of Electrical Engineering and Information Technologies, University of Naples Federico II, Via Claudio 21, 80125 Naples, Italy; ^3^Division of Gastrointestinal Medical Oncology, Department of Abdominal Oncology, Istituto Nazionale Tumori-IRCCS “Fondazione Giovanni Pascale”, Via Mariano Semmola, 80131 Naples, Italy; ^4^Division of Gastrointestinal Surgical Oncology, Department of Abdominal Oncology, Istituto Nazionale Tumori-IRCCS “Fondazione Giovanni Pascale”, Via Mariano Semmola, 80131 Naples, Italy; ^5^Divion of Radiotherapy, Department of Diagnostic Imaging, Radiant and Metabolic Therapy, Istituto Nazionale Tumori-IRCCS “Fondazione Giovanni Pascale”, Via Mariano Semmola, 80131 Naples, Italy; ^6^Division of Diagnostic Pathology, Department of Diagnostic and Laboratory Pathology, Istituto Nazionale Tumori-IRCCS “Fondazione Giovanni Pascale”, Via Mariano Semmola, 80131 Naples, Italy

## Abstract

To evaluate MRI for neoadjuvant therapy response assessment in locally advanced rectal cancer (LARC) using dynamic contrast enhanced-MRI (DCE-MRI) and diffusion weighted imaging (DWI), we have compared magnetic resonance volumetry based on DCE-MRI (*V*(DCE)) and on DWI (*V*(DWI)) scans with conventional T2-weighted volumetry (*V*(C)) in LARC patients after neoadjuvant therapy. Twenty-nine patients with LARC underwent MR examination before and after neoadjuvant therapy. A manual segmentation was performed on DCE-MR postcontrast images, on DWI (*b*-value 800 s/mm^2^), and on conventional T2-weighted images by two radiologists. DCE-MRI, DWI, and T2-weigthed volumetric changes before and after treatment were evaluated. Nonparametric sample tests, interobserver agreement, and receiver operating characteristic curve (ROC) were performed. Diagnostic performance linked to DCE-MRI volumetric change was superior to T2-w and DW-MRI volumetric changes performance (specificity 86%, sensitivity 93%, and accuracy 93%). Area Under ROC (AUC) of *V*(DCE) was greater than AUCs of *V*(C) and *V*(DWI) resulting in an increase of 15.6% and 11.1%, respectively. Interobserver agreement between two radiologists was 0.977, 0.864, and 0.756 for *V*(C), *V*(DCE), and *V*(DWI), respectively. *V*(DCE) seems to be a promising tool for therapy response assessment in LARC. Further studies on large series of patients are needed to refine technique and evaluate its potential value.

## 1. Introduction

Rectal cancer is a frequent malignancy in both men and women, accounting for 40.290 new cases in the USA in 2012 [1]. Despite the efforts done to introduce screening programs, most patients are diagnosed in a locally advanced stage of the disease (T3-T4, Nx, and Mx). Total mesorectal excision (TME) combined with preoperative radiation therapy and concurrent chemotherapy (pCRT) is the current standard for locally advanced rectal cancer (LARC) [2]. TME is associated with significant morbidity and functional complications, evolving conservative treatment strategies are being developed for patients with early rectal cancer at diagnosis and patients with significant/complete tumor regression after pCRT. A further conservative strategy has been to adopt a “wait and see” policy, omitting surgery when a complete clinical response is obtained after pCRT. This strategy has the advantage of reducing morbidity and provides a “true” organ-sparing approach, considering that sphincter preservation without adequate function has questionable benefit. CRT induces tumor downstaging and complete or partial pathologic responses through vascular changes and cell death [3]: a pathologic complete response (pCR) was verified in up to 24% of patients. A pCR is known to be associated with a favorable oncologic outcome, with regard to both recurrence and survival [4, 5]. Morphological MRI evaluation (mMRI) is considered the best available tool for LARC staging, allowing an accurate evaluation of the disease extent, up to and beyond and over the mesorectal fascia, and of the lymph node involvement [6]. However, there are some limitations in depicting the changes after CRT through morphological MRI alone. A favorable tumor response may not correspond to an appreciable tumor size reduction. MR imaging, like other morphologic imaging techniques (endorectal ultrasonography and computed tomography) is hampered by interpretation difficulties in assessing the presence of residual tumor within areas of radiation-induced fibrosis [6]. Studies are therefore focusing on the potential added benefit of functional and/or quantitative methods of MR image evaluation. Functional MRI visualizes underlying biological characteristics of tumors, adding a new dimension to the morphological information from conventional MRI. The combination of objective MRI biomarkers with detailed morphological MRI makes MRI a potentially powerful response measurement tool that provides comprehensive information on tumor heterogeneity and changes in heterogeneity as a result of treatment. Dynamic contrast-enhanced MRI (DCE-MRI) has proven useful in detecting residual tumor after CRT [7]. Previous studies have been investigated functional parameters derived by DCE-MRI dataset for noninvasive response assessment in various malignancies, including rectal cancer [8, 9]. Moreover, in various oncology fields, researchers have recently suggested that diffusion-weighted imaging (DWI) can potentially be used to identify biomarkers of treatment response [3]. These assertions are based on the fact that DWI could provide individual tumor apparent diffusion coefficient (ADC) increase rates during the course of CRT, which could reflect biological tumor changes.

DCE and DW MR imaging after CRT were shown to be more valuable than morphologic MR imaging to recognize pathological response from residual tumor, because on DCE and DW images, viable tumor remnants are more easily recognized, as they appear hyperintense compared with the low signal intensity (SI) of the surrounding non neoplastic tissue. Hence, it can be hypothesized that volumetry of the tumor that is based on SI characteristics on DCE or DW images may be more accurate than conventional MR volumetry to distinguish between complete and noncomplete responders.

With this study, we aim to determine the diagnostic performance of DCE and DW imaging for the assessment of a pathological response after CRT in patients with LARC by means of volumetric SI measurements (*V*(DCE) and *V*(DWI), resp.) and to compare the performance of DCE and DW imaging with volumetry on conventional T2-weighted MR images.

## 2. Materials and Methods

### 2.1. Patient Selection

Twenty-nine consecutive patients with a median age of 62 years (range 29–76 years) were enrolled in this prospective study, from March 2010 to November 2013. All patients had a biopsy-proven rectal adenocarcinoma. Endorectal ultrasonography, MRI of the liver and pelvis, CT of the abdomen and pelvis, and chest X-ray were used as staging procedures. Inclusion criteria were patients with clinical T4, nodal involvement, or T3 N0 with a tumor location of ≤5 cm from the anal verge, or a circumferential resection margin of ≤5 mm, defined by MRI. Exclusion criteria were inability to give informed consent, previous rectal surgery, and contraindications to MRI or to MR contrast media. All patients were enrolled within the phase I-II prospective trial described in [10] which was approved by the Independent Ethical Committee of our institution. They all gave written informed consent to participate in the trial.

### 2.2. Neoadjuvant Therapy

External radiation therapy was performed using a 3-field technique (one posteroanterior and two lateral fields). Standard fractions of 1.8 Gy/day to the reference point were given, 5 times a week up to a total dose of 45 Gy. All patients received biweekly bevacizumab at 5 mg/kg plus three biweekly cycles of oxaliplatin at 100 mg/m^2^ and raltitrexed at 2.5 mg/m^2^, on day 1, and levo-folinic acid at 250 mg/m^2^, and 5-Fluorouracil at 800 mg/m^2^ on day 2 [2, 10].

### 2.3. MRI Data Acquisitions

All patients underwent DCE-MRI examination before and after pCRT (90 days on average, range 86–94 days between the two MRI evaluations). Imaging was performed with a 1.5 T scanner (Magnetom Symphony, Siemens Medical System, Erlangen, Germany) equipped with a phased-array body coil. Patients were placed in a supine, head-first position. Mild rectal lumen distension was achieved with 60–90 mL of nondiluted ferumoxil (Lumirem, Guerbet, RoissyCdGCedex, France) suspension introduced per rectum [11]. Precontrast coronal T1w 2D turbo spin-echo images and sagittal and axial T2w 2D turbo spin-echo images of the pelvis were obtained. After that, axial DWIs were acquired (spin-echo diffusion-weighted echo-planar imaging [SE-DW-EPI]) at three *b*-values of 0, 400, and 800 sec/mm^2^. Subsequently, axial, dynamic, contrast-enhanced T1w, FLASH 3D gradient-echo images were acquired. We obtained one sequence before and ten sequences, without any delay, after IV injection of 2 mL/kg of a positive, gadolinium-based paramagnetic contrast medium (Gd-DOTA, Dotarem, Guerbet, RoissyCdGCedex, France). The contrast medium was injected using Spectris Solaris EP MR (MEDRAD Inc., Indianola, PA), with a flow rate of 2 mL/s, followed by a 10 mL saline flush at the same rate. Temporal resolution was 0.58 minutes, corresponding to 35 seconds (as reported in Table 1). Total acquisition time for precontrast and ten postcontrast sequences was 6.4 minutes. Then, sagittal, axial, and coronal postcontrast T1w 2D turbo spin-echo images, with and without fat saturation were obtained (Table 1). The axial images were acquired without any angulation. Axial T1-w pre- and postcontrast sequences were acquired at the same position as the T2-w sequence. MRI total acquisition time was around 40 minutes. Patients did not receive bowel preparation, antispasmodic medication, or rectal distention before any of the MR examinations.

### 2.4. Image Data Analysis

The MR images were evaluated on a picture archiving and communication system and were independently analyzed by two radiologists with years of specific expertise reading pelvic MR images. The observers were blinded to each other's results, the clinical patient data, and pathology reports. The readers calculated tumor volumes by manually tracing the tumor boundaries slice by slice on DCE-MRI derived images obtained subtracting the basal and the 5th post-contrastographic series (*V*(DCE)), on diffusion weighted image a *b*-value of 800 s/mm^2^ (*V*(DWI)) and on conventional T2-weighted images (*V*(C)).

Whole-tumor volume was then calculated considering the total number of pixel (slice by slice) and multiplying this by pixel size in mm^2^. On the T2-weighted images, tumor was defined as areas of isointense signal as compared with the relatively lower hypointense signal of the normal adjacent muscular rectal wall. On post-CRT T2-weighted MR images, areas of markedly low SI at the location of the primary tumor bed were interpreted as fibrosis. As the risk for residual tumor in these fibrotic areas is known to be ±50%, they were also included in the volumetric measurements [12]. On DCE-MRI data sets, measurements were performed on subtraction images considering basal signal and 5th postcontrastographic series. Area of hyper-intense signal compared with normal bowel wall or background of lower SI tissue, were considered as tumor. On the DW images, measurements were performed on high *b* value (800 sec/mm^2^) images and were based on a visual analysis. To avoid errors due to T2 shine effect only areas with high SI on high-*b* value images and low SI on ADC maps, when compared with the normal bowel wall or background of lower SI tissue on high-*b* value images, were considered tumor.

For each data sets (DCE, DWI, and T2-weigthed), the readers determined (a) pre-CRT tumor volume; (b) post-CRT tumor volume (TV); and (c) the tumor volume reduction ratio (Δ volume), which was calculated as follows: (TV_pre_ − TV_post_) × 100/TV_pre_, where TV_pre_ is pre-CRT tumor volume and TV_post_ is post-CRT tumor volume.

### 2.5. Surgical Approach and Evaluation of Pathologic Response

All patients underwent total mesorectal excision after completing pCRT. An anterior resection or an abdominoperineal resection was performed on the basis of the results of post-CRT restaging. The surgical specimens containing the tumor were processed and evaluated by a single pathologist who was not aware of the clinical and MRI findings. Specimens were examined according to the Sixth American Joint Committee on Cancer TNM staging system. The tumor regression grade (TRG) was evaluated according to the method of Mandard et al. [13, 14]. TRG 1 means a complete response with absence of residual cancer and fibrosis extending through the wall. TRG 2 is the presence of residual cancer cells scattered through the fibrosis. TRG 3 corresponds to the presence of fibrosis and tumor cells, with predominancy of fibrosis. TRG 4 indicates as fibrosis and tumor cells, with predominancy of tumor cells. TRG 5 is the absence of regressive changes. Patients with a TRG 1 or 2 score were considered as responders, whereas the remaining patients (TRG 3, 4, or 5) were classified as not responders [9].

### 2.6. Statistical Analysis

Tumor volume percentage changes of the responder and nonresponder groups were analyzed using the Mann-Whitney test. A Wilcoxon signed-rank test was used to compare pre- and post-CRT volumes. Receiver Operating Characteristic (ROC) curves were also used to compare the diagnostic performance of *V*(DCE), *V*(DWI) and *V*(C) reduction rates after pCRT. Area Under ROC Curve (AUC) were calculated [15] and optimal thresholds were obtained maximizing the Youden index [16]. Sensitivity, specificity, positive predictive value and negative predictive value were performed considering optimal cut-off values. Fischer exact tests were used to investigate if results were statistically significant. Interobserver agreement was also obtained using interobserver correlation coefficient (ICC) for continuous variables (0–0.20, poor agreement; 0.21–0.40, fair agreement; 0.41–0.60, moderate agreement; 0.61–0.80, good agreement; and 0.81–1.00, excellent agreement).

A *p* value < 0.05 was considered significant for all tests. All analyses were performed using Statistics Toolbox of Matlab R2007a (The Math-Works Inc., Natick, MA).

## 3. Results

Histopathologic analysis of the surgical specimen yielded the following findings: 5 patients had T0, 6 had T1, 14 had T2, 3 had T3, and 1 had T4 tumor. Five patients had TRG 1, 9 patients had TRG 2, 7 patients had TRG 3, and 8 patients had TRG 4.

Table 2 shows patient characteristics and volume changes assessed by the three volumetric methods: T2-w volumetric change, DCE-MRI volumetric change and DW-MRI volumetric change.  Table 3 shows median and standard deviation values of volumes assessed by tree volumetric approaches before and after treatment. Wilcoxon's test findings reported significant decreases in volumetric measures, between pre- and posttreatment, assessed with all methods (Table 3): *V*(C) median value decreased from 36.9 cm^3^ to 16.7 cm^3^; *V*(DCE) median value decreased from 30.9 cm^3^ to 16.5 cm^3^; *V*(DWI) median value decreased from 14.59 cm^3^ to 6.0 cm^3^ (*p* < 0.01).

**Figure 1:**
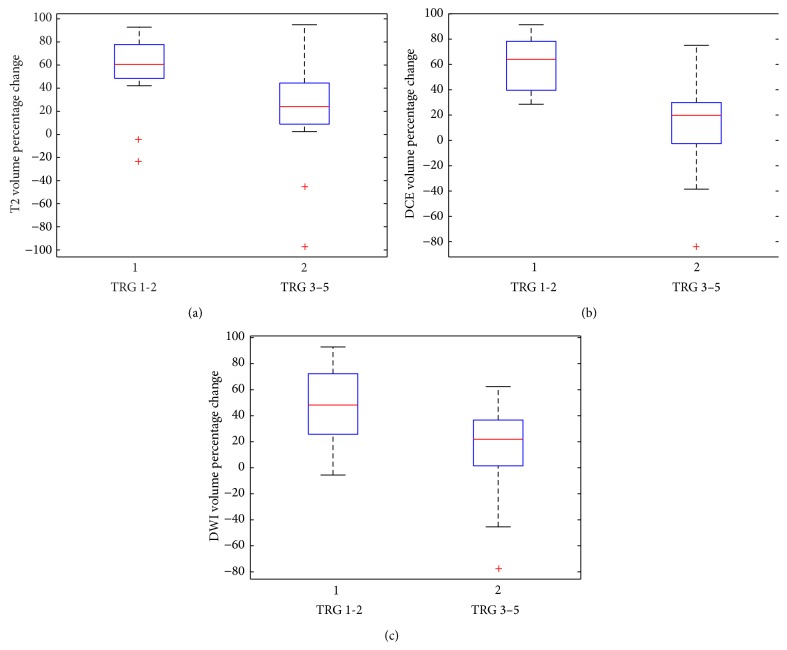
Boxplots of three volumetric approaches: (a) shows *V*(C) boxplot, (b) shows *V*(DCE) boxplot, and (c) shows *V*(DWI) boxplot. The middle line is the median value. The inferior and superior extremes of the box correspond to the first and third quartiles, respectively. The whiskers lines correspond to values within 1.5 times the interquartile range from the ends of the box. Outliner data beyond the ends of the whiskers are displayed with a + sign.

Statistically significant differences between responders and not responders, in the volumetric percentage changes were obtained by Mann-Whitney test for all volumetric approaches (Table 3) and these were visualized in boxplots (Figure 1).  Table 4 reports the performance of three volumetric methods in terms of sensitivity, specificity, and area under ROC. Optimal cut-off values were also reported. The results are statistically significant (Fisher test *p* < 0.01).

**Figure 2:**
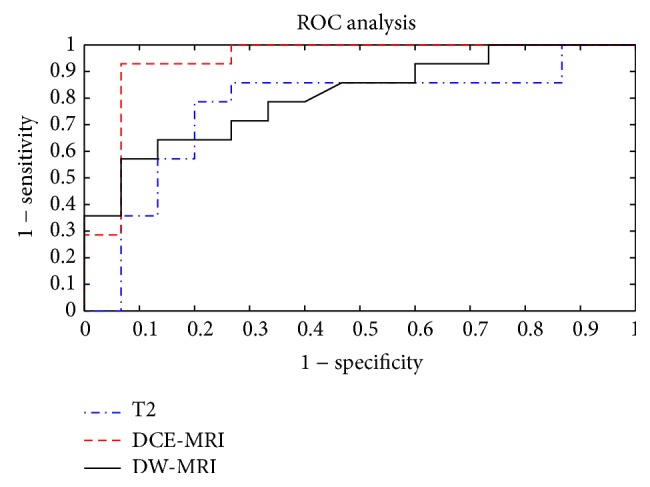
ROCs of three volumetric approaches (*V*(C), *V*(DCE), and *V*(DWI)).

According to TRG, DCE-MRI volume estimations change after pCRT showed best results in terms of specificity, sensitivity, and accuracy, respectively, of 86%, 93%, and 93%. *V*(C) showed a significant correlation between response and TRG with a sensitivity, a specificity and an accuracy, respectively, of 86%, 73%, and 79% (37% cut-off, AUC 0.76), and *V*(DWI) showed a sensitivity, a specificity and an accuracy, respectively, of 64%, 94%, and 76% (39% cut-off, AUC 0.81).  Figure 2 shows ROCs for three volumetric approaches. AUC of *V*(DCE) change is superior of AUCs of *V*(C) change and *V*(DWI) change resulting an increase of 15.6% and of 11.1%, respectively.

Interobserver agreement was 0.977 (95% confidence interval (CI) 0.954–0.989), 0.864 (95% CI 0.835–0.884), and 0.756 (95% CI 0.705–0.780) for *V*(C), *V*(DCE), and *V*(DWI).

## 4. Discussion

### 4.1. Synopsis of New Findings

The focus of this study was to clarify the association between manual tumor volume estimations obtained by T2-weigthed imaging, DWI, DCE-MRI images, and TRG to predict LARC response after pCRT. This study shows that manual tumor volume estimation using DCE-MRI can furnish better performance in terms of sensitivity, specificity, and accuracy. To the best of our knowledge, no prior studies have compared the performances of manual tumor volume measurements on T2-weighted (T2-w), DWI, and DCE-MRI images to predict LARC response after pCRT.

### 4.2. Comparisons with Other Studies

Tumor volume has been proven to be an important prognostic indicator for a variety of tumors, although the extra workload necessary to perform manual tumor volume measurements on MRI images have limited its applications in clinical routine. The goal to be achieved after pCRT for LARC is pathologic complete response. The latter could be assessed using the TRG, a pathological score widely considered as a potential tool to guide therapy in patients with LARC being an independent predictor of the likelihood of local recurrence, distant metastasis, and overall and disease-free survival [5]. Many approaches using MRI have been investigated to compare response after pCRT with TRG. The correlation shown between MR tumor volume estimation on T2-w images and the pCR has been the subject of many reports [17–19], even if conflicting results were reported: Kang et al. [20] showed a significant association with pCR for patients with a tumor volume reduction rate of more than 75%, whilst Kim et al. [21] observed no significant difference between patient with a pCR and those with residual disease. DW-MRI is an alternative technique potentially able to overcome part of *V*(C) limits, being capable of showing viable neoplastic tissue due to its reliability in detecting the restricted proton diffusion in hypercellular tissues with high nucleocytoplasmic ratio. According to some authors, the volume measurements obtained on post-CRT DW MR images were significantly more accurate than those obtained on post-CRT T2-w MR images to assess pathological complete response [22–25]. DCE-MRI is another technique that can provide functional information on tumor viability deriving tumoral neoangiogenic changes linked to contrast medium kinetics. Many authors described its potential advantages in predict response to therapy in different tumors [5–7], where neoformed tumor capillaries, being leaky, can determine a rapid gadolinium uptake, an early wash-out and overall shorter first pass, especially if compared with healthy tissues. Moreover, this kinetic behavior of contrast agent in DCE-MRI has been observed as correlated with some biomarkers expressed by highly vascularized tumoral tissues, such as the vascular endothelial growth factor expression and the microvessel density [7–9, 26]. Volumetric measure could be utilized in association with functional quantitative MRI parameters to increase their accuracy for tumor response therapy assessment. In fact, a recent study of Bajpai et al. [27] reported that apparent diffusion coefficient obtained on DW-MRI did not correlate with necrosis after chemotherapy in osteosarcoma but on adjusting for volume (apparent diffusion coefficient per unit volume); significant correlation was found and this appears to be a sensitive substitute for response evaluation in osteosarcoma. Future investigations could be done using these recent findings.

However, few authors have proposed a tumor volume estimation based on DCE-MRI images, using a manual or an automatic segmentation, mainly focusing their works on breast cancer [27, 28]. Moreover, to the best of our knowledge, no study has investigated the association between these volumes measured by DCE-MRI and TRG in LARC.

### 4.3. Study Limitations

*V*(C) showed a significant correlation between response and TRG with a sensitivity, a specificity and an accuracy, respectively, of 86%, 73%, and 79% (37% cut-off, AUC 0.76). Despite the excellent agreement between observers (0.977), understaging was present in 5 patients in which were assessed a decrease in volume >38% and a TRG of 3 or 4, confirming that T2-w volume assessment alone is not sufficiently accurate, being not able to identify between the persistence of viable tissue and the peritumoral fibrosis present after radiation therapy. Moreover, according to observers, was difficult to decide which fibrotic areas, remaining suspicious for tumor on T2-w images, should be included in the volume measurements and which should not. *V*(DWI) showed a sensitivity, a specificity, and an accuracy, respectively, of 64%, 94%, and 76% (39% cut-off, AUC 0.81) confirming the ability of *V*(DWI) in reducing the understaging observed with *V*(C) estimations. The agreement between observers was good (0.756) and both observers during segmentation easily and quickly recognized high-SI areas of “viable tumor” thus avoiding the inclusion of fibrotic areas that, due to their poor water content coupled with lower nucleocytoplasmic ratio, were not visible on either high *b*-value images and ADC maps. However, the low sensitivity of 64% obtained in our series reflects some intrinsic limits of *V*(DWI) measurements: during evaluation it is mandatory to remember that small bleedings or friable haemorrhagic/necrotic tissues can impact negatively on DW evaluations, causing field inhomogeneity due to susceptibility artefacts that, causing “enlargement” and “distortion” of “bright” areas on *b*-800 images, can make the tumor segmentation not accurate; the low spatial resolution with relative “small voxels” obtained, despite the large Field of View (FOV) used, can be a source of wide differences in estimated volumes causing tumor overestimation on post-CRT scans. Moreover, to reduce potential sources of false negative due to overestimation of residual tumor, a comparative evaluation with ADC maps is mandatory when areas of necrosis are identified and can be wrongly included in tumor being recognised like bright areas on images acquired with high *b*-values (≥*b*-800) due to the “T2 shine effect” [29]. *V*(DCE) showed best results with a sensitivity, a specificity, and an accuracy, respectively, of 86%, 93%, and 93% (34% cut-off, AUC 0.90) coupled with an excellent agreement between observers (0.864). Under staging, only in 1 patient was present that, although showing a great decrease in volume >37% on pCRT scan (75%), showed a TRG of 4. This false negative was probably due to the tumor appearance, which was mainly vegetant, with a great decrease in volume on *V*(DCE) measurements particularly on lumen side, whilst the persisting extramural viable tumor tissue was more easily detectable on T2-w and DW scans (as large spiculae in mesorectal fat-pad or high intensity areas on *b*-800 images). Only 2 patients were false positive: these patients constituted the main limit of *V*(DCE) segmentation. The choice of segmenting tumor on subtracted images obtained after 140s from contrast agent administration leaded to an inclusion of those areas in which inflammation phenomena were prominent (areas characterized by a continuous rise in SI followed by a stable intensity over the time) with an intrinsic potential overestimation, mainly on after pCRT scans. However, being *V*(DCE) focussed to identify “not effective” neoadjuvant treatments a false positive could be more tolerated than a false negative.

### 4.4. Clinical Applicability of the Study

Despite interesting results in terms of overall performance of *V*(DCE), currently, standard methods for tumor volume assessment based on manual delineation of volumes are time consuming. The mean time required to perform a volume measurement using multiple ROIs was approximately 10–15 minutes when pre-and post-CRT scans were considered and experienced radiologists employed, which is impractical. Therefore, semiautomated segmentation is needed.

## 5. Conclusions

In literature comparative studies between DCE-MRI, DW-MRI, and conventional MR volumetry are not present to assess therapy response evaluation after pCRT in LARC. In our series, manual segmentation of tumor volumes, made on DCE-MRI subtracted images, showed the best results in terms of sensitivity, specificity, and accuracy. When these results are validated in a larger prospective study and semiautomated software will be available they could be implemented in routine practice.

## Figures and Tables

**Table 1:** Pulse sequence parameters on MR studies.

Sequence	Orientation	TR/TE/FA (ms/ms/deg.)	AT (min)	FOV (mm × mm)	Acquisition matrix	ST/Gap (mm/mm)	TF
T1w 2D TSE	Coronal	499/13/150	2.36	450 × 450	256 × 230	3/0	3
T2w 2D TSE	Sagittal	4820/98/150	4.17	260 × 236	256 × 139	3/0	13
T2w 2D TSE	Axial	3970/98/150	3.48	270 × 236	256 × 157	3/0	13
SE-DW-EPI	Axial	2700/83	6.37	136 × 160	160 × 102	4/0	/
T1w FLASH 3D	Axial	9.8/4.76/25	0.58	330 × 247	256 × 192	3/0	/
T1w FLASH 3D	Axial	9.8/4.76/25	0.58 × 10	330 × 247	256 × 192	3/0	/
T1w 2D TSE	Sagittal	538/13/150	2.35	250 × 250	256 × 230	3/0	5
T1w 2D TSE	Coronal	538/13/150	2.52	250 × 250	256 × 230	3/0	5
T1w 2D TSE	Axial	450/12/150	2.31	270 × 236	256 × 202	3/0	5

Note: TR = repetition time, TE = echo time, FOV = field of view, FA = flip angle, ST = slice thickness, TF = turbo factor, and AT = acquisition time.

**Table 2:** Patient characteristics with volumetric change for individual patient.

Patient number	Age	pT	pN	TRG	*V*(C) change [%]	*V*(DCE) change [%]	*V*(DWI) change [%]
1	74	2	0	4	37,10	33,28	38,86
2	50	1	0	2	70,76	91,34	61,08
3	75	1	0	3	−96,50	29,88	36,67
4	54	1	0	2	61,22	67,40	−5,60
5	50	2	0	2	77,65	35,07	81,40
6	58	0	0	1	52,36	46,52	7,69
7	57	1	0	3	53,18	−3,71	62,42
8	67	0	0	1	80,34	70,57	72,33
9	70	1	1	2	59,99	39,59	46,72
10	75	2	0	2	77,81	78,21	22,49
11	78	2	1	3	8,85	−38,50	1,51
12	68	2	0	3	20,20	27,42	18,58
13	48	0	0	1	92,84	86,56	92,89
14	44	2	1	3	2,37	21,72	3,00
15	59	4	0	4	94,93	8,51	24,50
16	60	1	0	2	−4,52	28,52	39,50
17	63	2	0	3	21,73	13,52	−86,38
18	58	2	0	4	35,39	−2,57	43,52
19	44	2	0	4	66,01	34,08	22,63
20	63	3	1	4	28,05	3,26	−77,05
21	69	2	1	2	48,51	44,32	64,58
22	76	2	1	2	42,17	68,19	25,73
23	72	0	0	1	85,58	79,34	84,50
24	61	2	0	4	14,12	−83,35	−45,41
25	64	3	1	4	−45,75	20,64	21,25
26	58	3	0	3	44,51	18,94	28,28
27	76	2	0	2	50,72	60,61	36,40
28	77	2	0	4	13,16	75,04	−16,52
29	57	0	1	1	−23,73	12,45	49,65

Note: pT = pathological T stage; pN = pathological N stage; *V*(C) = conventional T2-weighted volumetry; *V*(DWI) = diffusion-weighted imaging volumetry; *V*(DCE) = dynamic contrast enhanced volumetry.

**Table 3:** Volume measures assessed by the three volumetric methods (*V*(C), *V*(DCE), and *V*(DWI)).

Tool	All (*n* = 29)	Responders (*n* = 14)	Nonresponders (*n* = 15)	*p* value
*V*(C)				
Pre-CRT [cm^3^]	36.9 ± 34.4	30.6 ± 22.6	38.4 ± 26.6	
Post-CRT [cm^3^]	16.7 ± 8.8	12.4 ± 6.9	20.6 ± 9.5	
Volume change [%]	44.5 ± 43.0	60.6 ± 33.2	21.7 ± 46.6	<0.001^*∗*^
*V*(DCE)				
Pre-CRT [cm^3^]	30.9 ± 29.1	31.4 ± 23.7	33.9 ± 25.4	
Post-CRT [cm^3^]	18.5 ± 8.1	11.1 ± 6.7	24.7 ± 10.8	
Volume change [%]	33.3 ± 38.4	64.0 ± 23.7	19.0 ± 36.9	<0.001^*∗*^
*V*(DWI)				
Pre-CRT [cm^3^]	14.6 ± 12.6	12.5 ± 10.1	15.8 ± 9.3	
Post-CRT [cm^3^]	9.0 ± 5.1	6.4 ± 4.4	7.9 ± 5.5	
Volume change [%]	28.3 ± 43.1	48.2 ± 30.0	21.2 ± 44.0	<0.001^*∗*^

Note: results are expressed as median value ± standard deviation.

^*∗*^Wilcoxon signed-rank test.

CRT, chemoradiation therapy; *V*(C), conventional T2-weighted volumetry; *V*(DWI), diffusion-weighted imaging volumetry; *V*(DCE), dynamic contrast enhanced volumetry.

**Table 4:** Performance of volumetric approaches (*V*(C), *V*(DCE), and *V*(DWI)).

	Sensitivity [%]	Specificity [%]	Accuracy [%]	Cut-off [%]	AUC
*V*(C) change	0.86	0.73	0.79	37.4	0.76
*V*(DCE) change	0.86	0.93	0.93	34.2	0.90
*V*(DWI) change	0.64	0.94	0.76	39.1	0.81

Note: *V*(C), conventional T2-weighted volumetry; *V*(DWI), diffusion-weighted imaging volumetry; *V*(DCE), dynamic contrast enhanced volumetry; AUC, area under ROC.
